# Comparison Between Strain and 2D Shear Wave Elastography of Testes in Healthy Dogs

**DOI:** 10.3390/ani15040498

**Published:** 2025-02-10

**Authors:** Francesca Del Signore, Roberta Bucci, Massimo Vignoli, Marco Russo, Camilla Smoglica, Andrea De Bonis, Andrea Rubini, Martina Rosto, Augusto Carluccio, Domenico Robbe, Salvatore Parrillo

**Affiliations:** 1 Department of Veterinary Medicine, University of Teramo, Piano d’Accio, 64100 Teramo, Italy; rbucci@unite.it (R.B.); csmoglica@unite.it (C.S.); adebonis@unite.it (A.D.B.); arubini@unite.it (A.R.); mrosto@unite.it (M.R.); acarluccio@unite.it (A.C.); drobbe@unite.it (D.R.); sparrillo@unite.it (S.P.); 2 Department of Veterinary Medicine and Animal Production, University of Naples, Federico II, 12, 80137 Naples, Italy; marco.russo@unina.it

**Keywords:** dog, testis, ultrasound, strain elastography, shear wave elastography

## Abstract

Sonoelastography is a rapid and non-invasive ultrasound-based technique to assess tissue elasticity. Interest in this technique’s application in veterinary medicine is progressively increasing, with preliminary reports also available to assess canine testicular parenchyma abnormalities. Since elastographic techniques are different and it is of paramount importance to standardize procedures, this work aimed to compare two different elastographic techniques, SE and 2D SWE, on the same testes and compare the data with the current literature. Thirty healthy canine testes were included, and complete B-mode, color and power Doppler examinations were performed on each one. SE was performed qualitatively through an elastogram evaluation and semiquantitatively comparing the parenchyma with peripheral skin. 2D SWE was performed and m/s measurements were taken. Each measurement was collected in the cranial, middle and caudal portions, considering the sections above and below the mediastinum separately. The healthy testes appeared to be intermediate in stiffness with differences in the various portions for SE and expressed a mean value from 1.3 m/s to 1.4 m/s for 2D SWE, consistent with the previous literature. Considering the technical specifics of the software for the two elastography methods, both of them are valuable in assessing canine testis stiffness and are potentially clinically appliable.

## Simple Summary

Sonoelastography is a rapid and non-invasive ultrasound-based technique to assess tissue elasticity. This work aimed to compare two different techniques, strain elastography and 2D SWE, on healthy canine testes, to provide a baseline for future studies and compare the data with the currently available literature. A total of 30 testes were included, with complete B-mode, SE and 2D SWE examinations. SE evidenced a difference between testes portions above and below the mediastinum, which was partially detected by 2D SWE. The results herein described are consistent with the literature on human and veterinary medicine, thus making these two techniques valid in clinical practice when taking into account their specific technical peculiarities.

## 1. Introduction

In canine species, testicular ultrasonographic imaging is an important diagnostic tool because it allows for the non-invasive assessment of the shape, size, position, margination and internal architecture of these structures and facilitates the study of vascularization. It is also possible to detect small lesions or those inaccessible by palpation and provides the details of testicular tissue that cannot be obtained by radiography [1,2].

However, even though the classic ultrasonographic examination is a very sensitive method for detecting neoplastic testicular lesions in particular, in many cases, it is impossible to assess the type of lesion based on the examination without diagnostic techniques, such as fine-needle aspiration cytology, testicular biopsy or histopathology [2,3].

In recent years, a rapid and non-invasive US-based technique named elastography has been introduced into clinical routine to investigate tissue abnormalities based on the different grades of stiffness expressed by investigated tissues affected by different types of disorders [4,5].

The most common sonoelastography techniques are strain elastography (SE), 2D shear wave elastography (SWE) and acoustic radiation force impulse (ARFI) elastography.

SE is based on measuring the grade of strain produced by the operator who manually executes rhythmic and regular compressions with the probe in the area of interest that is deformed proportionally to its elasticity; the elasticity can be visually assessed evaluating a color map or semiquantitatively comparing the relative deformation of the tissue with a reference one, with a specific parameter called the strain ratio (SR). A SR < 1 shows that the studied tissue is softer than the reference one and the opposite if the SR is >1 [6,7].

SWE, in contrast, employs a force created by US pulses to generate shear waves that spread faster in harder tissues, with m/s and kPa expressing the tissue elasticity without the influence of the operator compression [8]. The difference between 2D SWE and ARFI relies on the fact that the latter is based on short, high-intensity acoustic pulses to deform the tissue and create a static greyscale map in which different gray points correspond to different tissue elasticities codified by the shear wave (SW) velocity, while 2D SWE measures the SWs at several points within a larger field of view (FOV) and depicts them in a color-coded map (called an elastogram), superimposed on the gray-scale image. The advantages of this technique include the real-time visualization of a color quantitative elastogram superimposed on a B-mode image, enabling the operator to be guided by both anatomical and tissue stiffness information [8,9].

The potentially reduced need for more invasive diagnostic tests to assess tissue abnormalities is leading to a progressive increase in interest in the application of various sonoelastography techniques, with many reports available about elastography feasibility studies performed on the liver, kidney, lymph nodes, skin, muscles and tendons in both dogs and cats [10,11,12,13,14,15,16,17]. There is also greater interest in elastographic applications for canine testes, with reports available about healthy and diseased testes [1,18,19].

Healthy canine testes have been described as having a mean velocity of 1.65 ± 0.15 m/s with 2D SWE, and they expressed a higher stiffness in cases of infertility for SE and in cases of focal lesions with the ARFI technique [1,18,19].

The major limitation of using elastography on a large scale is the possibility of obtaining different measurements with different techniques, even with the same technique but set on different manufacturing sonographic machines; thus, a reference interval of measurements on healthy subjects with different software programs and sonographic machines is required [18].

The present paper aimed to assess normal SE and 2D SWE features on healthy canine testes and compare the results of the currently available literature with different software programs, exploring the feasibility of a different reference tissue for SR and assessing the possible agreement between the two techniques.

## 2. Materials and Methods

In this study, healthy male dogs were prospectively included from October 2023 to June 2024 with informed consent provided by the owners in all cases, including the Italian Army Canine Department in coordination with the Department of Veterinary Medicine.

The inclusion criteria were dogs in good general health assessed by a clinical examination, with no signs of reproductive disorders assessed by a complete semen quality examination following the current available literature [2] and with calm demeanor.

This bicentric study was conducted at Department of Veterinary Medicine of Teramo and Naples and approved by the Animal Ethic and Welfare Committee of the University of Naples under protocol N PG/2020/0063243 on 28 July 2020.

### 2.1. US Examination

Each dog underwent a complete testicular US examination with a Logiq S8 sonographic system (GE Healthcare, Milan, Italy) and an 8.5–10 mHz linear probe (9L, GE Healthcare, Milan, Italy); B-mode, color Doppler, power Doppler, SE and 2D SWE exams were performed with corresponding software using the same equipment by a single operator with 5 years of experience in US (FDS) and sonoelastography under the supervision of a board-certified radiologist with an ECVDI diploma (MV).

Both SE and 2D SWE were performed with the same frequency (10 mHz) and with technical settings provided by the software.

#### 2.1.1. B-Mode and Doppler Examination

**Figure 1:**
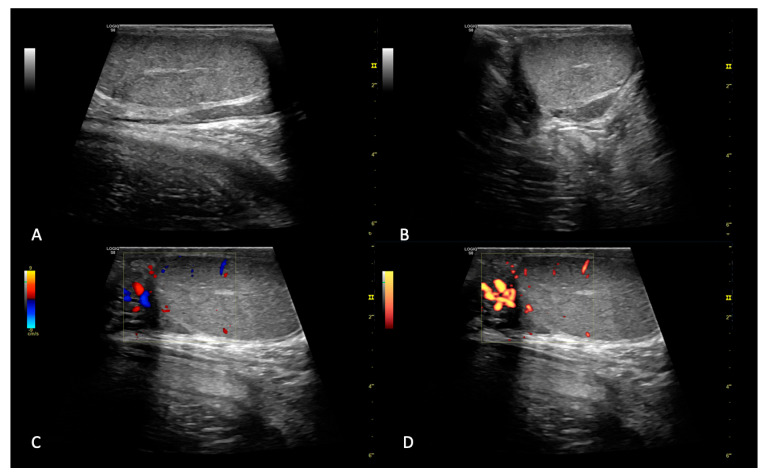
Example of B-mode and color Doppler examination. In panels (**A**,**B**), respectively, longitudinal and transverse testicle sections are reported; note the medium level of ecogenicity with a homogeneous, medium–coarse echostructure and smooth margins. In panels (**C**,**D**), respectively, color and power Doppler on a longitudinal testicular section are reported, with normal parenchymal and epididymal vascularization.

All the patients were manually restrained and kept in right lateral recumbency. B-mode, color and power Doppler exams were performed on both testes in longitudinal and transverse scans and were included only if they presented normal B-mode and Doppler features (Figure 1) [1,2].

#### 2.1.2. SE and 2D SWE Examination

Regarding the elastography examination, both SE and 2D SWE examinations were performed with the following elastography protocol, based on the current human and canine literature about SE and 2D-SWE applications.

SE was conducted with right and left testes scanned in the longitudinal orientation in order to collect for each testis a 4–5 s loop, from which several frames representing the positive or negative peaks of the strain graph could be obtained; three elastograms were collected with the color of elastography images unchanged between them [20].

**Figure 2:**
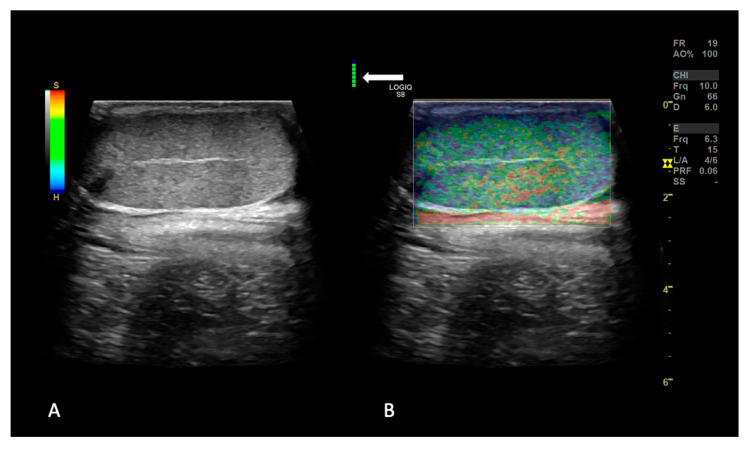
Example of strain elastographic image of a testis on the longitudinal section. The dual-mode, real-time, biplane elasto-mode shows the B-mode image on the left side (panel (**A**)) and elastographic image on the right side (panel (**B**)); the testis shows a predominantly green/blue pattern (type III). The acquired appropriate compression force and frequency of elastography are monitored using a color scale, with green color indicating the correct compression applied (white arrow).

The qualitative elasticity evaluation was performed including as much testicular parenchyma as possible in a longitudinal scan in a single FOV, with the stiffness evaluation performed using a color scale adapted from the current human literature. A score of 1 indicated a predominantly high-strain pattern in the testis with the entire testis predominantly shaded in red, scores of 2 to 4 indicated intermediate-strain patterns with the central and peripheral parts of the testis changing from red to green and blue, and a score of 5 indicated a low-strain pattern, with nearly the entire testis shaded in blue (Figure 2). The classification between 2, 3 and 4 was decided based on the amount of blue color recorded; if the predominant color was green followed by red without blue spots, it was considered a 2, if it had a predominantly green/blue pattern with still some red spots present, it was considered a 3, and if it had a blue/green pattern with predominance of blue and without red spots, it was considered a 4 [20].

**Figure 3:**
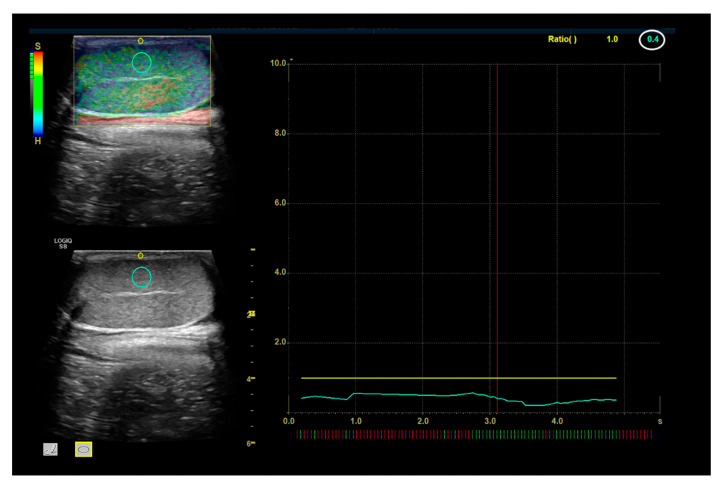
Example of testicular strain ratio measurement and evaluation. The light blue circle points to the examined testicular tissue (target tissue), with the yellow circle pointing to the skin considered as reference tissue. The result of this analysis is pointed out by the value within the white circle; values lower than 1 mean that the target tissue is softer than the reference one.

Then, the operator manually traced an area for elasticity index values (EIs) with a circular ROI of 6 mm placed on testicular parenchyma divided into cranial, middle and caudal portions. For each portion, the measurements were collected on dorsal and ventral sections (above and below the mediastinum) in order to avoid the testicle mediastinum. Then a circular ROI was traced on the skin (<3 mm based on the thickness) overlying the testes parenchyma ROI to obtain the strain ratio (SR). The strain ratio was calculated using the integrated software on the basis of strain histogram function; it was measured as the ratio of strain value for the organ parenchyma to that for the near-field skin within the same image and calculated as follows: strain ratio = mean strain value of organ parenchyma/mean strain value of skin (Figure 3) [20].

2D-SWE was then conducted acquiring a cineloop of 20 s on each section in a longitudinal orientation, then 5 elastograms were selected per each section and three ROIs of 6 mm were placed on the elastograms without overlapping them to collect measurements in m/s without including testicular mediastinum. The color scale of the elastogram ranged from red to blue (0.0–4.8 m/s), with red areas representing the softest tissue and blue areas representing the stiffest tissue.

**Figure 4:**
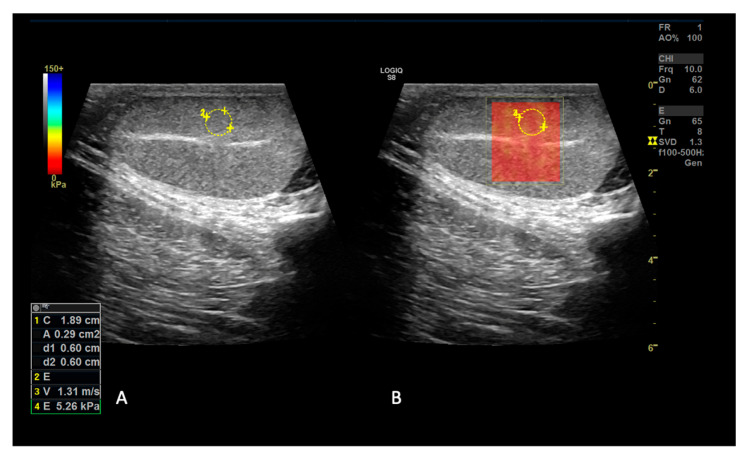
Example of 2D SWE examination of a testis on the longitudinal section. The B-mode longitudinal image of the normal testis is shown (**A**); the same image is visible with the superimposition of elastogram (**B**), whose colors are codified by the color bar present on the left side of the image. The elastogram appears homogeneously red, which states that the structure has low stiffness according to the color bar provided by the system. The circular ROI indicates where shear wave values expressed in m/s and kPa were measured, with results pointed out in panel (**A**).

Since it was not technically possible to scan the whole section with a single FOV, a rectangular FOV was set, respectively, on cranial, middle and caudal testes portions, acquiring m/s measurements both above and below the mediastinum as previously performed for SE (Figure 4) [21].

### 2.2. Statistical Analysis

For statistical purposes, the data analyzed were as follows:

- Qualitative map score for SE;
- EI and respective SR on each testicular portion for SE;
- m/s for 2D SWE.

For sonoelastography measurements, data regarding the three testes portions were labeled respectively cr for the cranial, md for the middle and cd for the caudal, with numbers 1 and 2 for the sections above and below the mediastinum, respectively.

Data regarding the sections above and below the mediastinum were analyzed separately, and the mean value was obtained to assess the difference in stiffness.

Data distribution was assessed, and data were expressed as mean ± standard deviation after being assessed for a normal distribution with Shapiro–Wilk test; *t*-test and ANOVA test were used to compare different groups of measurements, with a *p* < 0.05 indicating a statistically significant difference.

The color map distribution of the testes was included, and a comparison between left and right testes was made using the Chi2 test.

The agreement between the measurements was computed with intraclass correlation coefficient (ICC), classified as follows: 0.00–0.20 = poor; 0.20–0.40 = fair; 0.40–075 = good; and >0.75 = excellent [22].

Statistical analysis was performed with STATA 18 software.

## 3. Results

### 3.1. Patients

Fifteen dogs (30 testicles) met the inclusion criteria. The mean age of the subjects was 3.5 ± 1.2 years, and the breeds were German Shepherd (*n* = 8), Swiss Shepherd (*n* = 3), Malinois (*n* = 2) and mixed breed (*n* = 2), respectively.

A total of six testes from three dogs were excluded since their lack of compliance did not allow us to obtain accurate 2D SWE measurements.

### 3.2. Strain Elastography Evaluation

The qualitative color map evaluation evidenced that the majority of the testicles included were characterized by pattern 3, consisting of an intermediate level of stiffness with the central and peripheral parts of the testes changing from red to green and blue (Table 1).

Regarding EI, for the left testes, a significant difference was observed between the dorsal (1_cr) and ventral (2_cr) sections for the cranial and middle (1_md and 2_md) portions of the testes, while this difference was not observed for the caudal portion (1_cd and 2_cd) (Table 2); a significant difference was observed only in the cranial portion of the right one.

Considering the mean value between the sections above and below the mediastinum of each testis portion, there was no significant difference between the left and the right ones (Table 3).

Regarding SR, the same significant difference between the sections above and below the mediastinum of the testes segments was observed, respectively, in the cranial and middle segments for the left testis and in the cranial segment for the right one (Table 4).

Similar to the data collected for EI, the significant difference disappeared considering the mean value between the sections above and below the mediastinum (Table 5).

### 3.3. 2D Shear Wave Elastography

Analyzing the data obtained with 2D SWE, the measurement trend observed was similar, with differences observed for the left and right testes when single measurements were obtained, respectively, for the sections above and below the mediastinum in the middle portions (Table 6).

Similarly, the mean m/s values evidence no significant difference between each portion of the same testis and between the left and the right one (Table 7).

Finally, the ICC analyzed the reliability of each single measurement. Both SE and 2D SWE had excellent agreement (>0.9) for both the measurements collected on the single portions and the ones obtained from the mean values, thus showing the high uniformity of the measurements.

## 4. Discussion

In this work, SE and 2D SWE were performed on healthy canine testes in order to evaluate normal elastography data, explore a reference tissue that is different than the ones provided in the current literature and evaluate the evident technical peculiarities of the two techniques, which should be taken into account in clinical applications.

For qualitative SE, our data evidence that the testes appear to be characterized by a predominantly green/blue color, with the green color distributed in the central portion and the blue color mainly distributed at the edge of the parenchyma, with no difference between the left and the right one.

This is perfectly in line with human medicine, in which healthy testicles are characterized by an intermediate stiffness with this same color pattern distribution [23,24]; a peripheral soft ring is also reported and it is defined as a 3- rings pattern. However, authors refer to scrotal skin subcutaneous soft tissues as softer tissues than those in the actual testicular portion in elastograms [23]; this is different compared to canine species, whose skin is blue and hard as shown by sonoelastography, which is probably due to species differences due to the location of scrotal skin [15,17].

This is also in line with a previous report regarding SE for canine testes, in which infertile testes were stiffer than healthy ones [19].

Regarding the semiquantitative evaluation, both the EI of the testicular parenchyma and SR with skin were collected for every single testicular segment, as previously described.

The EI results herein collected evidenced a significant difference between the portions above and below the mediastinum, respectively, mostly evident in the cranial and middle portion of the testis, with the lower part significantly different compared to the upper one; moreover, even if there was not a statistically significant difference between the mean values, the measurements obtained in the central portion tend to be lower regardless of the position of the mediastinum.

This aspect has two possible explanations.

First of all, the depth at which the measurements are collected is a well-recognized influencing factor, since tissue attenuation decreases ultrasound signals intensity as a function of depth [7].

Moreover, the testicular parenchyma is characterized by a different anatomy, with the central part of the testes characterized by more seminiferous tubules and greater lymphatic and blood vascularization, as opposed to the higher tissue density in the rete testis area [18].

These data highlight the importance of collecting more measurements in different testicular parenchyma portions to obtain more thorough structural information, considering any evident differences, even if slight, due to tissue depth.

The same trend of the EI measurements was observed with SR, thus showing that the ratio reflects the differences in the testicular parenchyma observed with EI and is considerable as an alternative to the mediastinum as a reference tissue.

The previous report regarding SE for healthy and infertile testes provided the testicular mediastinum as a reference tissue.

The authors of the current work decided to use the skin as the reference tissue for two purposes: first, to compare data in the human literature with the data collected on canine species, and second, to obtain baseline data with a tissue external to the testes parenchyma in cases of testicular disorders, which can potentially affect the mediastinum too.

Hoverer, the choice of skin as a reference tissue deserves further clarification, since normally the reference tissue should be at the same depth of the target tissue and the ROI should be of the same size [25]. This is easily applicable for organs where a focal lesion is detected and a comparison is available with healthy tissue. This is not always applicable, especially in cases of diffuse parenchymal disorder or when there is a lack of reference tissues available at the same depth.

In human medicine, indeed, this is particularly true in the case of tendon disorders, in which the reference tissue is located above or below the tendon in most cases, including an external device as reference [26,27,28].

Also, in human medicine, to assess testicular parenchyma via SR, underlying skin has been used as a reference tissue [20]; the authors of the current paper are aware that the different depths of the two tissues may have an influence on the results. However, in the case of diffuse testes disorder, skin can be used as an alternative reference tissue to the testes mediastinum, since both EI and SR measurements collected on different testes portions have the same trend on different testicular portions, thus showing that the skin elasticity remains constant even if there are differences in testicular parenchyma stiffness.

Regarding the measurements collected in m/s for 2D SWE, Zappone and colleagues report 1.65 as the mean value for healthy testes, taking measurements with a different machine and 2D SWE software (Mindray DC-80A) but using the same procedure herein reported [18].

Unfortunately, a direct statistical comparison from the data herein reported and the data previously published is not reliable, but it is possible that, from a purely statistical point of view, a significant difference could be detected between the data herein reported and the ones from previous reports. The real question that should be answered is how this difference could really impact the clinical differentiation between healthy and diseased patients; based on the current available data, it could be assumed that values < 1.7 m/s can be considered as a reference for normal canine testis parenchyma.

Further studies on diseased canine testes will provide more information.

Compared to human medicine, these values are higher than the ones provided for healthy men; however, canine testes are characterized by a higher presence of fibrous tissue compared to that of human testes [1].

Interestingly, Zappone and colleagues evidenced no difference in 2D SWE values collected on different testicular portions above and below the mediastinum; this is partially in line with the results herein described, since a slight difference has been observed only in the central portion of both the right and left testes with 2D SWE.

This aspect further highlights the point already addressed: it is of paramount importance to standardize normal reference values with each software in order to avoid bias in the interpretation of results using different elastography software programs [18].

The first limitation of this study is the lack of a control group of diseased patients with whom to compare the measurements; however, this was considered as a successive step to be performed by the authors. With a baseline of consistent data on normal testis parenchyma, we compared our results to the literature already available, since sonoelastography can be highly operator-dependent.

Moreover, inter-observer reproducibility was not assessed in this paper; this was beyond the scope of the authors in this specific paper. The agreement between the measurements was only assessed considering the distribution of all the data and the eventual spread from a mean value, as values in a population of healthy dogs that are too different could lead to a reduced possibility of assessing the baseline for normal subjects.

However, especially for SE, the reproducibility of the technique can be limited by the influence of manual compression by a single operator [25]. For this reason, attention must be paid to the compression monitoring system provided by each software during the examination, but future studies on both healthy and diseased testes are necessary to assess the inter-operator reproducibility of SE.

Another limitation of the current study is the lack of histopathology; the structural and functional testis integrity was assessed through a clinical examination, US and semen analysis; however, the histopathology will be of paramount importance, especially in cases of testes lesions, to compare the collected data.

In the case of healthy dogs, testes should be assessed after castration, but none of the patients included underwent surgery during the study herein described.

Finally, the demeanor of the patients must be taken into account since the movement of the patient is a major obstacle to obtaining accurate measurements, especially for 2D SWE; indeed, three patients were excluded since only SE was performed and it was not possible to collect reliable 2D SWE measurements.

## 5. Conclusions

US elastography is a rapid and non-invasive technique able to assess tissue stiffness. Both SE and 2D SWE are valuable tools able to assess the elasticity of testicular parenchyma in healthy dogs. SE seems to reflect more clearly possible anatomical differences in the normal testicular parenchyma and skin is a valuable reference tissue when performing semiquantitative evaluations. Technical differences must be properly considered to correctly interpret sonoelastography results. More detailed data are necessary on diseased testes to confirm these results.

## Figures and Tables

**Table 1:** Color map data distribution for SE. Note that the majority of the testes included were characterized by pattern 3, followed by pattern 4.

Qualitative Evaluation	Right Testes		Left Testes		*p*-Value
	Freq.	Percent	Freq.	Percent	
1	0/15	0%	0/15	0%	0.622
2	0/15	0%	0/15	0%	
3	12/15	78.6%	13/15	85.7%	
4	3/15	21.4%	2/15	14.3%	
5	0/15	0%	0/15	0%	

**Table 2:** EI for each portion of the left and right testes. Note the significant difference above and below the mediastinum in the cranial and middle portions of the left testes and in the cranial portion of the right testes.

EI	Left Testes					*p*-Value
	Mean	Stand. Error	Standard Dev.	95% IC		
1_cr	1.35	0.23	0.87	0.85	1.85	0.01
2_cr	2.34	0.27	1.03	1.75	2.94	
1_md	1.39	0.19	0.73	0.97	1.82	0.004
2_md	2.51	0.32	1.21	1.81	3.21	
1_cd	2.24	0.32	1.22	1.53	2.95	0.62
2_cd	2.45	0.33	1.26	1.72	3.18	
**EI**	**Right Testes**					***p*-Value**
	**Mean**	**Stand. Error**	**Standard Dev.**	**95% IC**		
1_cr	1.45	0.25	0.94	0.911	2.00	0.01
2_cr	2.4	0.23	0.86	1.89	2.90	
1_md	1.83	0.31	1.18	1.14	2.51	0.62
2_md	2.03	0.31	1.18	1.34	2.71	
1_cd	2.07	0.36	1.37	1.27	2.86	0.28
2_cd	2.20	0.30	1.13	1.54	2.86	

**Table 3:** Mean EI for left and right testes. No significant difference was evident between the right and the left testes.

EI	Left Testes					*p*-Value
	Mean	Stand. Error	Standard Dev.	95% IC		
cr	1.84	0.23	0.86	1.34	2.34	0.977
md	1.95	0.22	0.83	1.47	2.44	
cd	2.34	0.29	1.11	1.70	2.99	
**EI**	**Right testes**					***p*-Value**
	**Mean**	**Stand. Error**	**Standard Dev.**	**95% IC**		
cr	1.92	0.20	0.77	1.48	2.37	0.16
md	1.93	0.29	1.10	1.29	2.56	
cd	2.13	0.31	1.17	1.45	2.81	

**Table 4:** EI for each portion of the left and right testes. Note the significant difference between the sections above and below the mediastinum in the cranial and middle portions of the left testes and in the cranial portion of the right testes.

SR	Left Testes					*p*-Value
	Mean	Stand. Error	Standard Dev.	95% IC		
1_cr	0.35	0.05	0.20	0.23	0.47	0.009
2_cr	0.65	0.07	0.29	0.48	0.82	
1_md	0.31	0.03	0.13	0.23	0.38	0.004
2_md	0.55	0.07	0.26	0.40	0.71	
1_cd	0.53	0.07	0.28	0.37	0.70	0.57
2_cd	0.59	0.07	0.29	0.42	0.76	
**SR**	**Right testes**					***p*-Value**
	**Mean**	**Stand. Error**	**Standard Dev.**	**95% IC**		
1_cr	0.34	0.05	0.19	0.22	0.45	0.02
2_cr	0.56	0.05	0.21	0.44	0.68	
1_md	0.35	0.05	0.20	0.23	0.48	0.50
2_md	0.41	0.06	0.24	0.27	0.55	
1_cd	0.45	0.07	0.26	0.30	0.61	0.61
2_cd	0.49	0.07	0.27	0.33	0.65	

**Table 5:** Mean SR of right and left testes. No significant difference was evident between the right and the left testes.

SR	Left Testes					*p*-Value
	Mean	Stand. Error	Standard Dev	95% IC		
cr	0.50	0.05	0.21	0.37	0.63	0.85
md	0.43	0.04	0.16	0.33	0.52	
cd	0.56	0.07	0.26	0.41	0.721	
**SR**	**Right testes**					
cr	0.45	0.04	0.16	0.35	0.54	0.104
md	0.38	0.05	0.21	0.26	0.51	
cd	0.47	0.06	1.11	0.33	0.62	

**Table 6:** Values of m/s for left and right testes. Notice the significant difference between parenchyma above and below mediastinum in the middle portion.

m/s	Left Testes					*p*-Value
	Mean	Stand. Error	Standard Dev.	95% IC		
1_cr	1.40	0.03	0.14	1.31	1.48	0.10
2_cr	1.31	0.04	0.15	1.22	1.40	
1_md	1.35	0.02	0.10	1.29	1.41	0.03
2_md	1.25	0.03	0.12	1.18	1.32	
1_cd	1.47	0.05	0.19	1.35	1.58	0.16
2_cd	1.36	0.04	0.18	*1.26*	1.47	
	**Right testes**					***p*-Value**
	**Mean**	**Stand. Error**	**Standard Dev.**	**95% IC**		
1_cr	1.42	0.05	0.20	1.30	1.53	0.29
2_cr	1.35	0.05	0.19	1.23	1.46	
1_md	1.40	0.04	0.17	1.30	1.50	0.02
2_md	1.27	0.03	0.13	1.19	1.35	
1_cd	1.47	0.04	0.16	1.38	1.57	0.09
2_cd	1.37	0.03	0.14	1.29	1.45	

**Table 7:** Mean m/s values for left and right testes. No significant difference was detected between the left and right testes.

m/s	Left Testes					*p*-Value
	Mean	Stand. Error	Standard Dev.	95% IC		
cr	1.35	0.03	0.13	1.28	1.43	0.11
md	1.30	0.02	0.10	1.24	1.36	
cd	1.42	0.04	0.18	1.31	1.52	
**m/s**	**Right testes**					***p*-Value**
	**Mean**	**Stand. Error**	**Standard Dev.**	**95% IC**		
cr	1.38	0.04	0.18	1.28	1.49	0.65
md	1.33	0.03	0.14	1.25	1.42	
cd	1.42	0.04	0.15	1.33	1.51	

## Data Availability

All the data available are included in the main text.
